# Numerical Analysis and 1D/2D Sensitivity Study for Monolithic and Laminated Structural Glass Elements under Thermal Exposure

**DOI:** 10.3390/ma11081447

**Published:** 2018-08-16

**Authors:** Marcin Kozłowski, Chiara Bedon, Dániel Honfi

**Affiliations:** 1 Department of Structural Engineering, Faculty of Civil Engineering, Silesian University of Technology, 44-100 Gliwice, Poland; marcin.kozlowski@polsl.pl or marcin.kozlowski@construction.lth.se; 2 Division of Structural Mechanics, Department of Construction Sciences, Faculty of Engineering, Lund University, SE-221 00 Lund, Sweden; 3 Department of Engineering and Architecture, University of Trieste, 34127 Trieste, Italy; 4 RISE Research Institutes of Sweden, 501 15 Gothenburg, Sweden; daniel.honfi@ri.se

**Keywords:** structural glass, laminated glass, experiments, finite element (FE) numerical modeling, one-dimensional (1D) models, two-dimensional (2D) models, thermal loading, material properties, thermal performance assessment, sensitivity study

## Abstract

Glass is largely used in architectural and engineering applications (i.e., buildings and vehicles) as a structural material, especially in the form of laminated glass (LG) sections. To achieve adequate and controlled safety levels in these applications, the well-known temperature-dependent behavior of viscoelastic interlayers for LG sections should be properly accounted for during the design process. Furthermore, the materials’ thermomechanical degradation with increases of temperature could severely affect the load-bearing performance of glass assemblies. In this context, uncoupled thermomechanical finite element (FE) numerical models could represent a robust tool and support for design engineers. Key input parameters and possible limits of the FE method, however, should be properly calibrated and assessed, so as to enable reliable estimations for the real behavior of glazing systems. In this paper, FE simulations are proposed for monolithic (MG) and LG specimens under radiant heating, based on one-dimensional (1D) and two-dimensional (2D) models. A special attention is focused on thermal effects, being representative of the first step for conventional uncoupled, thermomechanical analyses. Based on experimental results available in the literature, FE parametric studies are discussed, giving evidence of limits and issues due to several modeling assumptions. In particular, careful consideration is paid for various thermal material properties (conductivity, specific heat) and thermal boundaries (conductivity, emissivity), but also for other influencing parameters like the geometrical features of samples (thickness tolerances, cross-sectional properties, etc.), the composition of LG sections (interlayer type, thickness), the loading pattern (heat transfer distribution) and the presence of additional mechanical restraints (i.e., supports of different materials). Comparative FE results are hence critically discussed, highlighting the major effects of such influencing parameters.

## 1. Introduction

**Figure 1:**
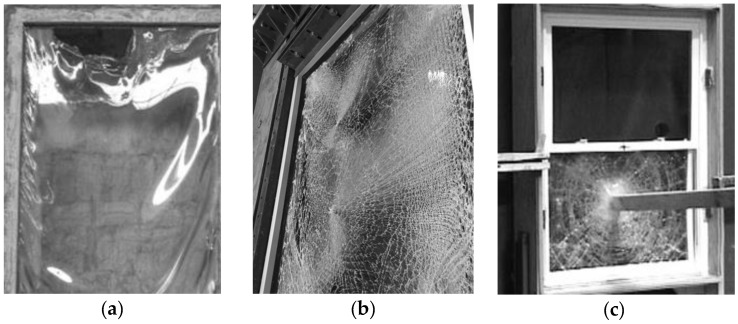
Examples of cracking and failure mechanisms in glass windows under (**a**) fire, (**b**) blast loading and (**c**) debris impact (photos reproduced and adapted from [7], CC BY 4.0, Copyright © 2017 Chiara Bedon).

Glass is increasingly used in buildings as a structural material for load bearing components like columns, beams and fins, plates for roofs and facades, as a major effect of aesthetic-related benefits [1,2,3]. The structural role of glass is also getting important in other fields, such as the automotive industry where LG windshields might contribute to the overall crasworthiness of vehicles [4,5]. However, the structural performance of glass systems acting as constructional components in buildings, under loading and boundary conditions of technical interest for safe design purposes, still requires investigations. Major issues are related to the material’s intrinsic features, including the thermophysical and mechanical properties and their sensitivity to ambient and loading conditions. As such, special care should be spent for extreme design actions that could derive from man-made and/or natural hazards, including severe temperature variations and fire, see for example Figure 1 and [6,7,8,9].

The performance of glass under thermal heating attracted attention of several research studies since the 50s, due to the consistent use of glass panels in windows and fenestrations. However, most of those investigations are related to the experimental assessment of thermal shock effects in ordinary, soda lime silica glass elements, while only limited studies and tests are currently available for the thermophysical and mechanical characterization of this constructional material. A minor part of these investigations is then related to the experimental and/or numerical analysis of composite glass systems and assemblies under combined thermomechanical loads [7,10]. The effect of special coatings and protective films, to improve the thermal stress resistance of glass specimens, has been also studied especially to reduce the effects of possible bridge phenomena in facades. In general, the aim of most of these projects consisted in the assessment and improvement of thermal and energy performance for glazing windows and fenestrations. In [11], for example, preliminary experimental tests have been reported for insulated glass specimens with silicone film coatings, for solar heating protection. No marked benefits, however, were noticed in the critical regions of the samples (i.e., close to the edges and in vicinity of the framing systems). Advanced CFD simulations were presented in [12], aimed at assessing the energy performance and indoor thermal comfort of glass curtain walls under the effects of a radiant floor heating system. Special care was spent for downdraft phenomena and prevention. In [13], preliminary thermomechanical numerical studies have been reported for glass–Glass Fiber Reinforced Polymer (GFRP) sandwich modular units for facades. Even in presence of ordinary design loads of limited magnitude, the investigation highlighted the importance of combined thermomechanical considerations for the performance assessment for the given structural systems.

Compared to existing research efforts, this paper focuses on the performance evaluation of glass under thermal exposure, based on FE numerical models and past experimental tests [14,15,16]. As a further extension of the research studies reported in [16], in particular, simplified, one-dimensional (1D) models and more refined, two-dimensional (2D) shell models are considered [17], including parametric analyses and sensitivity studies, so as to capture the effect of some key parameters. Special care is dedicated, in this investigation, to the thermal response of selected MG and LG samples, being representative of the first step for uncoupled, thermomechanical FE simulations conventionally in use for the structural analysis and performance assessment of building components and load-bearing systems. There, the predicted temperature scenarios have in fact a fundamental role for the overall thermomechanical response of a given structural sample, especially glass, being responsible of combined effects due to thermal and mechanical loads that should be properly accounted at the design stage.

As shown in the paper, careful consideration should be first spent for the input material characterisation, to account for temperature effects. However, a certain sensitivity is expected also from other key parameters, like boundaries (both thermal and mechanical), loading pattern, as well as size effects, that 1D models can only roughly describe due to their intrinsic basic assumptions.

Numerical analyses are hence discussed for selected MG and LG specimens under radiant heat flux, so to assess the accuracy and potential of both 1D and 2D FE models. To this aim, Section 2 briefly reports some basic aspects for structural glass under thermal loading, giving evidence of some major features that should be properly accounted. Past reference experiments are then presented in Section 3, including a description of major FE assumptions for the herein implemented 1D and 2D models. Based on the FE parametric results summarized in Section 4, some preliminary recommendations are then provided. Given the potential of 2D assemblies, compared to geometrically simplified 1D models, the effects of different thermal and mechanical boundary conditions are further emphasized, including sensitivity studies aimed to capture the effects of heat transfer distributions, or possible contact regions for the given glass samples (i.e., with additional mechanical restraints).

## 2. Material Properties and Temperature Effects

### 2.1. Basic Properties

Glass is a material characterized by a MOE in the range of 70 GPa [18], and by a typical brittle elastic tensile behavior, with limited effective strength. Although the conventional thermal or chemical pre-stressing processes can increase the reference characteristic tensile strength of float AN glass (with 45 MPa the nominal value [18]), by a factor of about two (for HS) or even three (in the case of FT elements), the occurrence of both local or global failure mechanisms due to possible tensile peaks should be properly prevented.

LG panels and systems, in this context, represent the majority of structural glass applications in buildings or automotive industry, etc., being typically characterized by the presence of two (or more) glass layers and one (or more) intermediate bonding foils, acting in the form of flexible shear connections. Common interlayers are composed of thermoplastic films like PVB, but can include also SG or EVA components. In general, the shear stiffness of these bonding layers is relatively low and depends on several conditions (i.e., time loading, temperature, humidity, etc.), see for example [1,2]. Further issues in the load-bearing performance of glazing systems are related to thermal loading, such as temperature gradients due to daily exposure and/or fire.

### 2.2. Specific Heat and Thermal Conductivity

**Figure 2:**
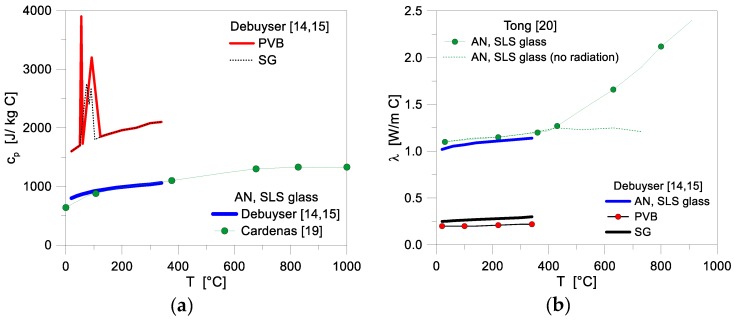
Thermal properties of glass and common interlayers, as a function of temperature. (**a**) specific heat *c_p_* and (**b**) conductivity *λ* (figures adapted from [7]).

Specific heat and thermal conductivity represent, from a numerical point of view, the first key input parameters for the performance assessment of glass systems under thermal exposure, especially when composite resisting sections consisting of LG panels are examined. However, literature references are rather limited for standard SLS glass in use for engineering applications, and even more for the bonding interlayers. In this research study, input features were taken from previous studies, see Figure 2 and [14,15,19,20].

### 2.3. Thermal Shock Performance

Generally speaking, thermal shock resistance in glass is conventionally estimated as a function of an allowable temperature gradient Δ*T* that the glass panels can withstand. Such a temperature gradient—being affected by several geometrical and mechanical parameters, including the glass panels thickness, possible pre-stressing and/or edge treatments, etc.—can be accounted according to prEN thstr:2004 provisions [21] and generally lies in the range from 22 °C up to 200 °C, see Table 1. As far the limit values of gradients in Table 1 are not exceeded, the glazing component should be able to withstand possible thermal shock phenomena.

Besides such a list of conventional values of interest for design purposes, a large number of experimental studies related to the thermal performance and resistance of glass has been focused on thermal breakage (see for example some recent studies in [22,23,24,25,26]), being representative of the major reason of glass cracking for windows and fenestrations. Given the number of existing research studies, however, the topic still requires investigations, since even counterposed findings can be derived from past research projects [7].

### 2.4. Temperature Dependence of Mechanical Properties of Glass Systems

The thermomechanical FE numerical analysis of glass systems is a complex task, due to a combination of several aspects. There, the material thermo-physical properties and their sensitivity to temperature exposure should be properly accounted, see Section 2.1 to Section 2.3. Another key aspect for structural assessment purposes, however, is then represented by the variation with temperature of the mechanical features (i.e., stiffness and resistance) of the same materials, namely represented for glass by the MOE and the tensile strength.

In this regard, given the combined effects of both thermal and mechanical related aspects, the typical load-bearing performance and failure mechanism for a given glass assemblies under elevated temperatures is markedly different from the expected behavior at room temperature [10]. The elastic properties of standard SLS glass at elevated temperatures, in this regard, have been extensively studied by several authors after the 50s, giving evidence of some typical observations and phenomena that should be properly accounted at the design stage (see for example [7], where a literature review is reported). The numerical uncertainties can increase when LG assemblies are investigated, in place of MG components [7]. An additional issue for the numerical analysis of glazing systems under thermomechanical loading is then represented by the possible interaction of glass elements (both MG or LG) with different materials—including mechanical supports, restraint effects, and/or possible local detailing—since even minor influencing parameters might play and important role (see for example [10]). In this research study, following [16], thermal phenomena are considered only for MG and LG samples, as a first major outcome of an ongoing research study. Accordingly, the variation of materials mechanical properties with temperature is temporarily disregarded and will be properly assessed in a future extension of the project.

## 3. Experimental and Numerical Studies

### 3.1. Reference Tests

Debuyser et al. presented in [14,15] a set of experimental results for MG and LG specimens, bonded together by PVB or SG interlayers, composed of float AN glass. The experiments included radiant panel tests, with measurements of the thermal properties for glass and interlayer materials. *B* = 285 mm wide × *H* = 185 mm high (*L* the total thickness) MG and LG specimens with different cross-section (16 samples, in total) were exposed to a heat source generating a relatively constant radiative heat flux. The glass samples were fixed to a wooden frame and subjected to thermal loading. In terms of loading measurements during the experiments, a central heat flux gauge was used to monitor the transmitted flux behind each glass sample; a side gauge (in plane with the surface of glass panels) was also used, since directly exposed to radiation like the samples; the reflected heat flux was finally captured by a heat flux meter, placed behind the radiant panel.

**Figure 3:**
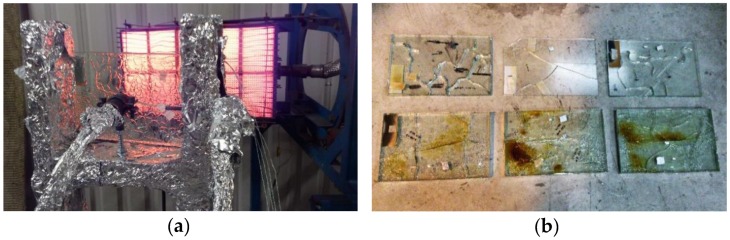
Thermal experiments on MG and LG specimens: (**a**) test setup (with an exposed LG sample) and (**b**) typical damage scenarios for the failed specimens, at the end of the radiant heating tests (Copyright @ Dániel Honfi).

The typical experimental setup is presented in Figure 3a, where the supporting frame, the central heat flux gauge and the side heat flux gauge are covered with aluminum foil. A standardized procedure was taken into account for the experiments, so as to ensure mostly comparable thermal boundary and loading conditions for all the samples. Until a rather stable radiant heat flux was achieved, in particular, an insulating board was placed in front of the radiant panel. The insulating board was removed only after mounting the MG and LG samples within the frame, so that each specimen could be exposed to the assigned radiant heat flux. At the end of the typical test, the glass panel was removed from the frame and the incident heat flux was measured (at the same position of the removed glass surface). In doing so, the temperature evolution due to the imposed heat exposure was continuously monitored. Different TC configurations were used, to record the temperature-time curves on both the exposed and the unexposed side of each sample. In the case of some LG specimens only, additional TCs were installed between the glass plies, being embedded in the interlayer foils before the lamination process. To protect the TCs from radiation (both on the exposed surface of glass and within the interlayer foils), small pieces of aluminum tape were used for shielding.

In terms of measurement of the key thermal properties for AN glass and PVB or SG interlayers, the conductivity, diffusivity and volumetric heat capacity values were experimentally determined. Based on the so collected test results, the emissivity of glass was found to be moderately dependent on the considered spectrum. In the case of LG samples, for both the interlayer type materials, the heat absorption was also experimentally derived.

In accordance with earlier research efforts, a relatively limited resistance and low thermal performance of MG and LG specimens was generally observed, due to the premature occurrence of thermal cracks in AN glass, as well as to the poor thermal reaction of the bonding interlayers. The typical pattern of cracks in the glass panels and bubbles in the bonding layers (formed in the melting and evaporating foils) can be observed in Figure 3b, for selected LG samples.

To develop and validate the reference thermal FE model herein discussed, three test setups and results were selected from [14,15], within the total set of samples. The tests denoted as “T2” and “T4”, in particular, were chosen since representative of 10 mm and 15 mm thick MG panels, respectively, whereas the “T5” sample was a LG panel (6 + 10 + 6 mm the nominal thickness of glass layers), with 0.76 mm thick PVB films. Besides the thermophysical material parameters (see Section 3.2), the main input of the FE models was represented by the heat flux to which the MG and LG panels were exposed. Since the incident heat flux was not directly measured at the time of the past experiments, for the sake of simplicity, the thermal histories measured by the heat flux meters at the side of the samples were reasonably used in this numerical study, since slightly underestimating the real heat flux values in time (in turn, reflectance was neglected in the calculations).

**Figure 4:**
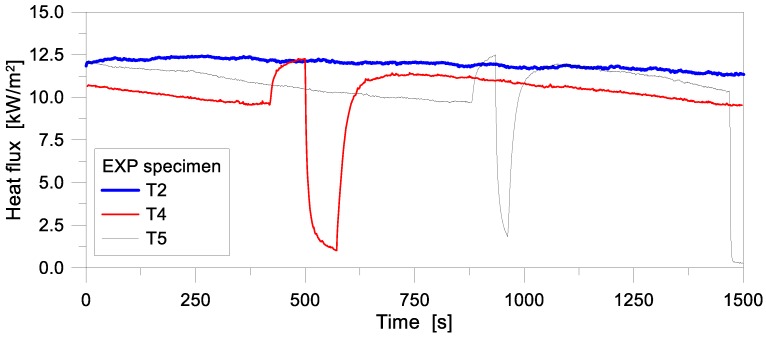
Experimentally measured heat flux histories (at the side of samples, see Figure 3a) for the specimens T2, T4 and T5.

In this regard, the test results of side heat flux measurements for the selected T2, T4 and T5 samples are presented in Figure 4. There, it can be seen that the imposed heat flux was not stable during the experiments, but slightly decreasing with time, especially for the T4 and T5 samples. Sudden drops for the heat flux histories of the T4 and T5 samples can be also noticed after ≈600 s and ≈1000 s of testing, being caused by unintentional shutting off of the radiant panel. After these incidents, the radiant panel was re-powered and the tests continued up to 1500 s. The final drop for the T5 specimen, in this regard, was due to the introduction of the insulation board in front of the sample, so as to remove the glass panel from the wooden frame, at the end of the experiment.

### 3.2. One-Dimensional (1D) Numerical Modlling

The main aim of the numerical study herein summarized was to investigate the temperature evolution through the thickness of MG and LG samples subjected to the heat flux histories of Figure 4.

**Figure 5:**
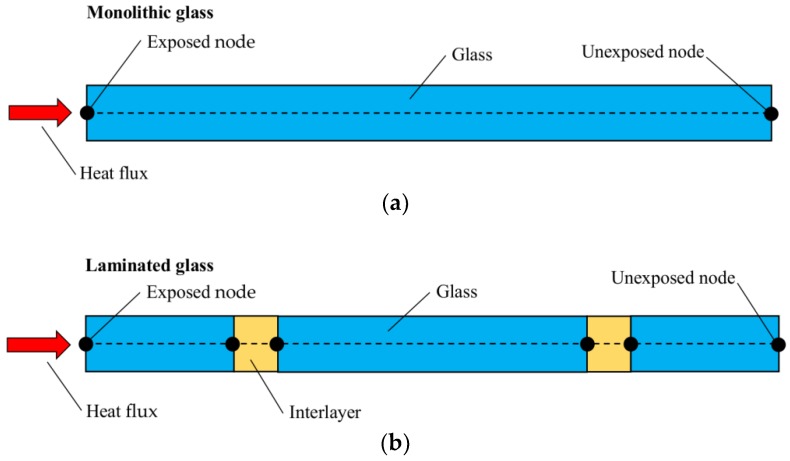
Schematic representation of the reference 1D heat transfer model for (**a**) monolithic and (**b**) laminated glass specimens (ABAQUS).

The typical one-dimensional (1D) heat transfer model, similar to those presented in [14,15] was created using the commercial computer software ABAQUS/Standard [17] and consisted of two-node, one-dimensional diffusive heat transfer elements (DC1D2 type, from ABAQUS library), see Figure 5.

Within a so-assembled 1D model, the complex heat transfer phenomena within a given panel were modelled in the form of equivalent heat conduction and convection and radiation at the surfaces where glass is in contact with the air. The through-the-thickness absorption and emission, in particular, were assumed lumped at the front and backside surfaces of glass. In accordance with Figure 5a, the differential equation governing the so defined 1D problem within the glass thickness (i.e., 0 < *x* < *L*, with *x* = 0 for the exposed surface) was then given by:

(1) $$\frac{\partial T}{\partial t}=\left(\frac{\partial^{2}T}{\partial x^{2}}\right)\frac{\lambda }{\rho \cdot c_{p}}$$

where *λ* denotes the effective thermal conductivity, *ρ* is the density of glass, *c_p_* is its specific heat capacity (all the parameters are dependent on the temperature *T*).

At the exposed surface (*x* = 0), the radiant heat flux to the specimen *q_in_* (including the effects of transmittance, absorptance and reflectance) and the heat flux from the specimen *q*_*out*,1_ (including the effect of convection and emission through radiation) was taken into account in a simplified way, that is:

(2) $$-\lambda \left(\frac{\partial^{2}T}{\partial x^{2}}\right)+q_{in}-q_{out,1}=\rho \cdot c_{p}\frac{\partial T}{\partial t}$$

with *q_in_* given as a time series (alternatively, in similar problems, *q_in_* can be defined as a constant value) and *q*_*out*,1_ can be calculated according to Equation (4).

At the unexposed surface of glass (*x* = *L*) the heat emission was considered in the form of an additional heat flux term (*q*_*out*,2_), representing the convective and radiative heat transfer between glass and the surrounding air. The governing equation, in this latter case, was given by:

(3) $$-\lambda \;\left(\frac{\partial^{2}T}{\partial x^{2}}\right)-q_{out,2}=\rho \cdot c_{p}\frac{\partial T}{\partial t}$$

Both the terms *q*_*out*,1_ and *q*_*out*,2_ are defined as:

(4) $$q_{out}=h\cdot \Delta T+\varepsilon \cdot \sigma \cdot (T_{s}^{4}-T_{air}^{4})$$

In Equation (4), *h* denotes the convective heat transfer (or film) coefficient (see also Equation (7)), ∆*T* is the temperature gradient between the glass surface (*T_s_*) and the ambient air (*T_air_*), at a given time instant, *ε* is the emissivity of the surface, *σ* is the Stefan-Boltzmann constant. The above description refers to MG specimens according to Figure 5a; however, similar principles were used to account for the effects of interlayers, in the case of LG models agreeing with Figure 5b.

In terms of FE model calibration, following Equations (1)–(4), special care was spent for some input parameters. The thermal properties of glass and interlayers, such as conductivity and specific heat, were taken from literature (Section 2.2). According to Figure 5, the thermal exposure was then simulated by applying a concentrated heat flux to the exposed node of each FE assembly, in the form of a time-varying heat flux amplitudes derived from Figure 4. As a predefined condition for both the exposed and unexposed sides of each sample, an initial ambient temperature of 20 °C was considered, being well representative of the past laboratory conditions. Moreover, the following physical constants were taken into account for the reference FE models:

- an emissivity coefficient for glass surface equal to ε = 0.97,
- the Stefan–Boltzmann constant was set to *σ* = 5.67 × 10^−8^ W/m^2^K^−4^, with
- an absolute zero temperature of −273.15 °C.

A Fortran script user-subroutine was finally used to define the thermal boundary conditions between the external nodes of each FE assembly and the surrounding environment. This included a convective heat transfer coefficient (*h*) dependent on the varying temperature of the exposed and unexposed nodes (see also [27]).

Given a conventional heat transfer analysis, the *h* coefficient is dependent on the fluid properties (thermal conductivity, density and viscosity), flow parameters (velocity and nature of the flow) and the geometry of the sample (dimensions and angle of the flow). It can be expressed in terms of Grashof and Prandtl dimensionless groups that allow the physical properties of the fluid, the flow velocity and nature of convection, to be taken into account. The Grashof dimensionless group *G_r_* is usually expressed as:

(5) $$G_{r}=\frac{g\cdot l^{3}\cdot \beta \cdot \left(T_{1}-T_{0}\right)}{\nu^{2}}$$

where *g* is the gravitational acceleration constant (9.81 m/s^2^), *l* is the flame height (0.185 m, in this study), *β* is the coefficient of air expansion (3.41 × 10^−3^ K^−1^), *T*_0_ is the initial (ambient) temperature and *T*_1_ is the current temperature, while ν is the kinematic viscosity (1.51 × 10^−5^ m^2^/s). For the FE analyses herein presented, the air properties at 20 °C were derived from [28]. The Prandtl dimensionless group *P_r_* is then conventionally expressed as:

(6) $$P_{r}=\frac{\nu }{\alpha }$$

where *α* is the air thermal diffusivity (2.11 × 10^−5^ m^2^/s) and *ν* is defined in Equation (5).

The *h* coefficient is hence calculated as the product of Prandtl and Grashof numbers, and for a vertical plate with natural, laminar convection is given by:

(7) $$h=\frac{k\cdot 0.59\cdot {\left(G_{r}P_{r}\right)}^{1/4}}{l}$$

where *k* is the thermal conductivity of air (0.026 W/mK). Typically, *h* takes values in the range of 5–50 W/m^2^K for natural convection [28,29].

Based on the reference 1D models schematized in Figure 5, a set of FE analyses was carried out on the selected T2, T4 and T5 samples. In doing so, the sensitivity of 1D estimations to some input parameters was numerically assessed. Regarding the MG samples, in particular, the effects of varying emissivity and film surface coefficient were first investigated. Later on, additional parametric studies were focused on the variation of the nominal glass thickness, including manufacturing tolerances (i.e., ±0.5 mm for 15 mm thick glass panels, see [30]). In terms of LG specimens, different thicknesses for the PVB interlayer were also taken into account. An overview of the 1D parametric configurations discussed in this paper is summarized in Table 2.

### 3.3. Two-Dimensional (2D) Numerical Modelling

As a further extension of the 1D research study, 2D models allowing for a more accurate modelling of boundary and loading conditions (both thermal and mechanical) were successively taken into account. In this paper, the T2 monolithic glass sample (10 mm thick, AN glass) was selected for the sensitivity study.

**Figure 6:**
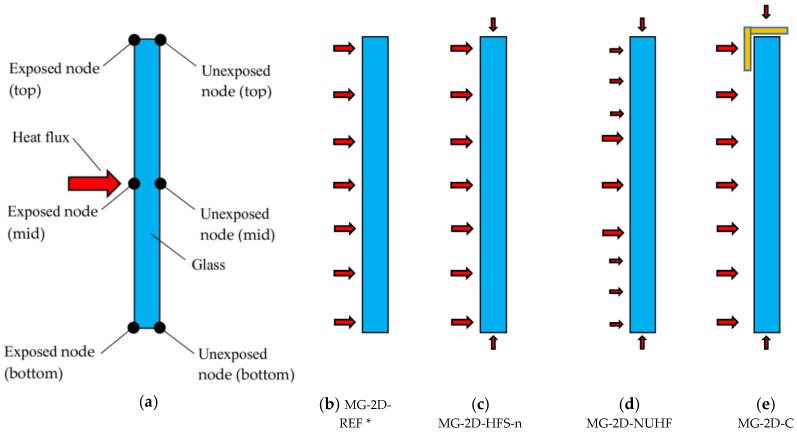
Schematic representation (front view) of 2D heat transfer models (ABAQUS): (**a**) location of the reference control points for the thermal performance assessment, with (**b**–**e**) different boundary and loading configurations for the FE parametric study. * = reference 2D model for the parametric study.

As in the case of 1D systems described in Section 3.2, the typical 2D numerical model was created in ABAQUS/Standard [17] to represent the middle cross-section of the T2 sample (10 mm wide × 185 mm high), see Figure 6a. The FE assembly hence consisted in 8-node quadratic, two-dimensional diffusive heat transfer quadrilateral elements (DC2D8 type, from ABAQUS library). Given a regular mesh pattern, the reference size of 2D shell elements was set to 1 mm, based on preliminary sensitivity studies not included in this paper, for sake of clarity. Such a choice ensured rather stable estimations of temperature histories in the thickness of the samples, as well as limited modelling and computational costs for the so assembled FE systems (less than 1900 shell elements and 6000 DOFs).

The thermal and mechanical properties of glass were assigned as in the case of the 1D models described in Section 3.3. Accordingly, the emissivity coefficient for glass and its temperature-dependent, convective surface, heat transfer coefficient were kept fix. In the sensitivity study, a set of FE configurations inclusive of four different boundary and loading conditions were considered, see Figure 6b–e, so as to assess their effects on the overall thermal performance of the selected samples. Special attention is spent, in this paper, for the discussion of FE parametric results from the T2 monolithic panel. The first examined configuration, see Figure 6b, represents an extension of the reference 1D model for the T2 specimen, and includes a heat flux-time history on the exposed (front) surface of glass only. As such, it is taken into account within the set of 2D parametric analyses for comparative purposes only, towards the corresponding 1D assembly.

Later on, for the second FE configuration, see Figure 6c, an additional heat flux was applied at the top and bottom edges of the T2 sample, so as to describe the real thermal boundary conditions of the experimental specimens discussed in Section 2. Such a loading condition was numerically taken into account with the aim at assessing the influence of different thermal exposure scenarios on the response of glass samples, at selected control points (i.e., in mid-span and top/bottom edge points of the middle cross-section). Given the uncertainties on the actual thermal exposure for the top/bottom faces of the tested specimen, however, as well as the lack of test measurements in support of the FE modeling, the heat flux at the top/bottom edges of the 2D models according to Figure 6c was numerically assumed as a ratio of the nominal flux histories of Figure 4, and to correspond—within the parametric investigations—to 5, 15 and 25% respectively of the nominal one. As in the case of Figure 6b, a uniform distribution of thermal fluxes was considered for each one of the 2D exposed edges.

The third configuration schematized in Figure 6d, in this regard, was successively taken into account for assessing the effect of nonuniform heat flux distributions on the glass surfaces. During the past experiments, it was in fact observed that the imposed heat flux was not uniform through the set of test samples (i.e., Figure 4), as well as on the exposed (front) face of each one of the glass samples, with progressively reduced heat flux amplitudes when moving from the center of the radiant panel towards its lateral regions (see [14,15]). Accordingly, the FE systems of Figure 6d were subjected to nonuniform heat flux distributions in time, on their front side. The fourth numerical configuration schematized in Figure 6e, finally, included a 5 mm thick steel clamp able to stabilize the specimens during the experiments, so as to schematically reproduce the mechanical restraints of the test samples within the wooden frame (see Figure 3a). At the glass-to-steel interface, in accordance with the past experiments, a thermal insulation layer (i.e., rigid wool fibre) with 3 mm the nominal thickness was also modeled. Given the specific mechanical boundaries for the FE configuration of Figure 6e, the imposed heat flux was kept uniform on the full exposed face of glass, as well as on the top/bottom edges, for direct comparative purposes towards the reference model (Figure 6b). According to the set of 2D models schematized in Figure 6, a more detailed overview of the FE parametric configurations herein discussed is summarized in Table 3.

## 4. Discussion of FE Numerical Results and Assessment towards the Past Experiments

A first assessment of 1D and 2D numerical predictions was carried out by taking into account the temperature distribution and evolution at the centre of the glass samples, in accordance with the available experimental data. In the figures reported in Section 4.1 and Section 4.2 for 1D and 2D models respectively, according to the labels of Figure 5 and Figure 6a, continuous lines are used to represent the temperature history at the FE node directly exposed to the imposed heat flux (“Exp”, in the following), while dashed lines are used for the unexposed node, on the backside of the samples (“UnExp”).

### 4.1. One-Dimensional (1D) Numerical Modelling

**Figure 7:**
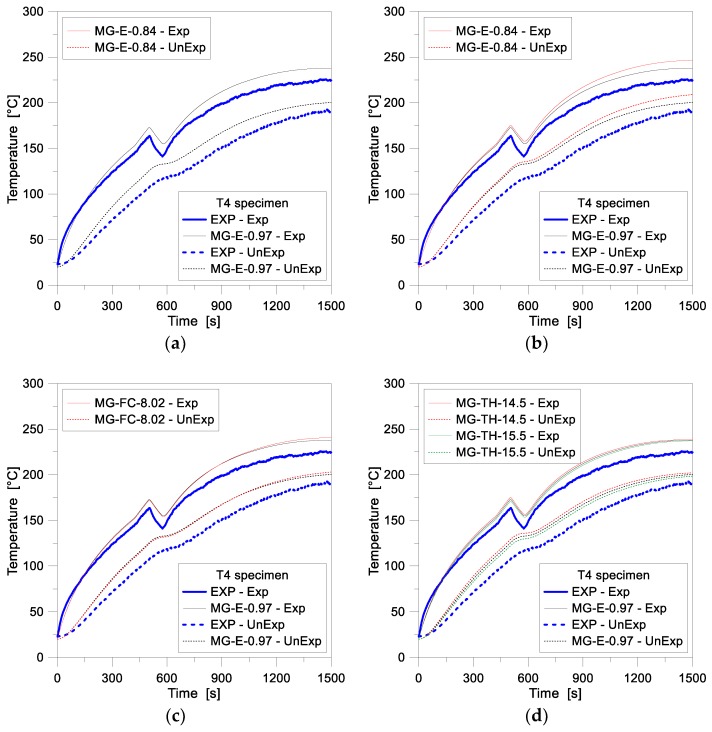
Temperature history comparisons for the MG specimen T4, as obtained from the FE parametric analyses (ABAQUS, 1D) and the past experimental test. FE results from (**a**) the reference 1D model and (**b**–**d**) the sensitivity study.

Figure 7 presents a comparison of numerical and experimental results for the MG specimen T4. According to Table 2, the figure illustrates the T4 thermal response, as obtained from variations in some key input features of glass, such as (i) emissivity, (ii) film surface coefficient (constant or temperature dependent values, respectively) and (iii) glass thickness.

In general, the FE numerical results were observed to slightly overestimate the experimental data, see Figure 7a–d. For the reference 1D model of Figure 7a, as well as for the FE parametric models in general, a close correlation was found for the Exp node especially at the beginning of the collected temperature histories, rather than at the later stage of the analyses (where the FE temperature values presented, in any case, less than 12% scatter, with respect to the test measurements). For the UnExp node of the same FE models, through the overall simulation time, the numerical results were indeed found to overestimate the experimentally measured temperatures, by approximately 10% the test values.

For all the comparative plots of Figure 7, at ≈500 s of thermal loading, a drop of temperature can be also observed, which was caused by the sudden shutting off of the radiant panel (see Section 2 and the T4 heat flux history of Figure 4). This phenomenon, as expected, proved to be more evident for the Exp node of the parametric FE models (≈25–30°). The UnExp node, even in presence of a relatively limited thickness for the T4 glass panel, was less affected by such a drop in the assigned loading history, due to the thermal inertia of the T4 glass volume, hence resulting in a less pronounced variation of the calculated temperature-time history. In terms of sensitivity of FE estimations to input parameters, see Table 2 and Figure 7b,c, minor effects due to variation of glass emissivity or film surface coefficient were found, and such an outcome applies especially to the early stage of the analyses. A change in the emissivity value resulted, up to ≈800 s of thermal loading, in less than 2% the calculated scatter between the parametric FE plot and the reference 1D model, see Figure 7c. For the final loading phase (>800 s), an average increase up to 4% was numerically observed, with respect to the reference assembly, with higher temperature measurements for lower glass emissivity values. In terms of film coefficient, see Figure 7b, a mostly negligible variation of temperatures (less than 1%) was indeed predicted, when replacing the US input with a constant value.

In Figure 7d, finally, the effects of glass thickness variations are shown. The temperature variations, compared to the reference 1D model, were found to be directly proportional to the glass panel thickness, both at the Exp and UnExp nodes, with an average scatter of ±0.6% and ±1.5% on the front and backside of the T4 sample.

**Figure 8:**
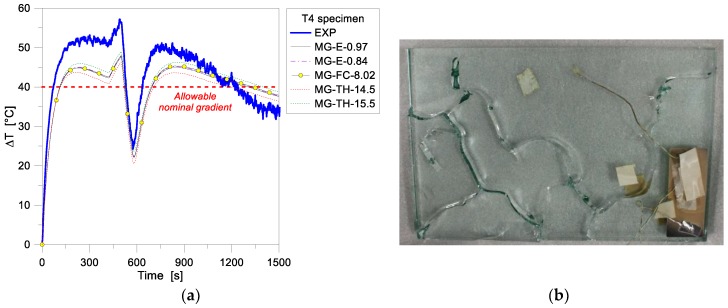
(**a**) Temperature gradient comparisons for the MG specimen T4, as obtained from the FE parametric analyses (ABAQUS, 1D) and the past experimental test, with (**b**) experimental crack pattern at the end of the test (reproduced from [14] with permission from Elsevier, Copyright license n. 4404210422661, August 2018).

Figure 8a, in this context, presents the temperature gradient Δ*T* calculated from the past experimental data, as well as obtained from the parametric FE simulations on the T4 sample, being representative of the temperature scatter, in time, between the Exp and UnExp nodes of the specimen. As shown, compared to test predictions, the numerical results were observed to underestimate the experimental values until ≈1200 s of exposure, while the opposite effect was found for the following time instants, up to the conclusion of the simulations. Since the temperature gradient is strictly related to potential failure of glass due to thermal shock phenomena (see Table 1), careful attention should be spent on this aspect. Worth of interest, in this regard, is that the experimentally measured and numerically simulated Δ*T* values were found to be much larger than the allowable temperature gradient given by the prEN thstr:2004 document (40 °C for 15 mm thick AN glass panels with polished edges).

At the time of the past experiment, the T4 sample gave evidence of severe cracks in the glass surface, see Figure 8b. The time instant of first glass fracture in the test, however, is not available for comparative purposes. From a qualitative point of view, given the FE estimations of Figure 8a and the sensitivity of glass mechanical properties to temperature variations [7,10], it is also expected that the overall uncoupled thermomechanical analysis of the same sample could also result in crack propagation. At the current stage of the FE study, see Figure 8a, emissivity and film coefficient modifications proved to have negligible effects on the Δ*T* values, as also in accordance with Figure 7 (with up to 1.3% the measured scatter). A higher sensitivity of the collected FE results was observed when varying the glass thickness (with ±7.2% the effect of product tolerance values on the nominal thickness of the sample).

**Figure 9:**
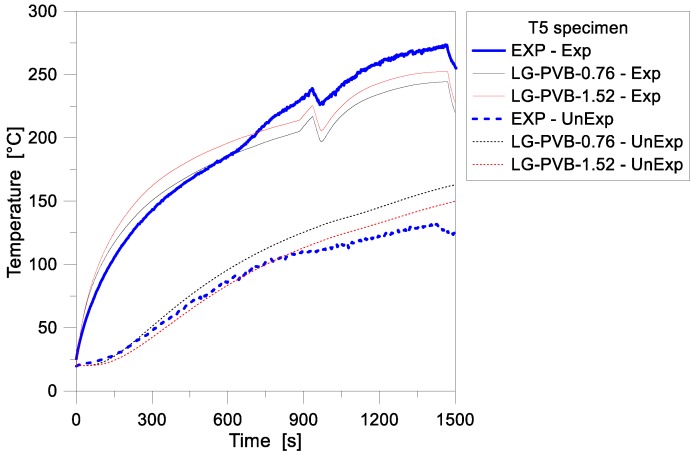
Temperature history comparisons for the LG specimen T5, as obtained from the FE parametric analyses (ABAQUS, 1D) and the past experimental test.

**Figure 10:**
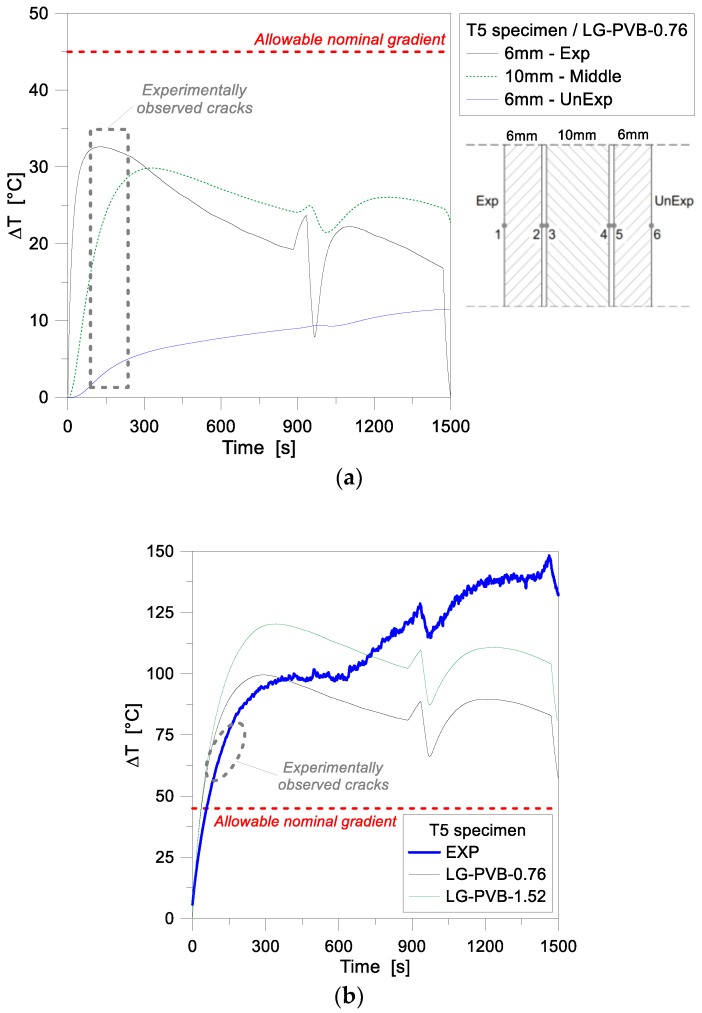
Temperature gradient comparisons for the LG specimen T5, as obtained from the FE parametric analyses (ABAQUS). (**a**) Numerical estimations for separate glass layers and (**b**) absolute temperature gradient for the full LG sample.

As far as LG specimens are taken into account in the FE investigations, further interesting conclusions can be derived on the reliability and accuracy of 1D models. Figure 9, for example, presents a comparison of numerical and experimental results for the T5 sample. The FE analyses, in this case, were specifically focused on the influence of varying the thickness of the PVB interlayer foils (with 0.76 mm the nominal value for the T5 specimen and 1.52 mm a numerical value of technical interest in the design of glazing systems). As in the case of the T4 specimen briefly discussed in Figure 7 and Figure 8, much better agreement with the past experimental values was observed for the initial stage of the FE analysis, for the model with nominal cross-sectional features (0.76 mm thick foils). This includes the temperature evolution at both the Exp and UnExp model nodes, with respect to the test. During the past experiment, similarly to the T4 sample, a sudden shutting off of the radiant panel took place. This accidental drop can be clearly perceived in the recorded time-temperature histories of Figure 9, at ≈950 s of loading. Major effects were experimentally and numerically perceived at the Exp node of the sample, as also in accordance with the T4 observations. In this latter case, the presence of multiple layers for the T5 sandwich cross-section typically resulted in a progressively increasing protection level for the middle and unexposed glass layers, respectively. Such a finding can be also perceived from comparative plots of Figure 10, where thermal gradients are proposed for the same sample.

As shown in Figure 9 and Figure 10, in addition, the parametric FE simulations emphasized that even small variations in the thickness of the bonding PVB foils can typically result in increased temperatures at the Exp node, and reduced temperatures at the UnExp node, compared to the reference geometrical configuration, as a direct effect of the added thermal inertial for the composite section. In terms of recorded temperature gradients, finally, the TCs for the past T5 sample were unfortunately mounted at the external surfaces (front and backside) of the panel only, thus no direct comparison of test data with the numerically predicted gradients in each glass ply can be performed. In any case, the FE models can offer some useful background, for a further extension and interpretation of the experimental results.

Figure 10a presents in fact the numerical temperature gradients for each ply, as obtained for the T5 assembly. The exposed surface, as shown, obviously heats up fastest, and a high temperature gradient (up to ≈35 °C) is achieved at the early stage of the simulation (≈130 s). The middle glass ply, being protected from direct radiant heating, shows Δ*T* values of slightly lower magnitude (≈30 °C the maximum value) and a certain delay in the temperature increase (≈250 s of loading for the gradient peak), compared to the exposed glass ply. At the same time, the middle glass layer further insulates the unexposed ply, which shows Δ*T* values that are in the order of 50% of the previous glass layers, and present a rather smoothed, linear trend. In Figure 10a, according to Figure 9, a sudden drop of Δ*T* values (up to 15°) for the exposed ply can be then observed, at approximately 950 s of the simulation. This finding, being related to the previously discussed experimental accident, is reduced to a minimum for the middle glass layer (≈8 °C), and fully vanishes in the temperature history for the unexposed ply.

**Figure 11:**
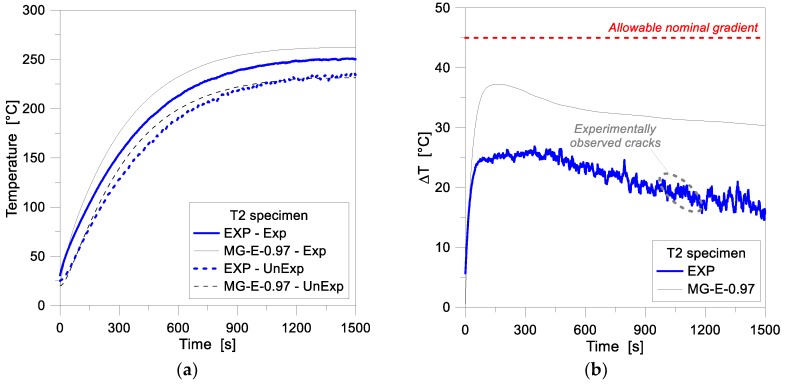
(**a**) Temperature history and (**b**) gradient comparisons for the MG specimen T2, as obtained from the FE analyses (ABAQUS, 1D), and the past experimental test.

According to standards, see Table 2, the thermal fracture is conventionally ensured in a given LG assembly as far as the allowable nominal gradient for the weakest ply is not exceeded. In the case of the T5 sample, the experimental crack was observed to initiate after ≈200 s of exposure. In this regard, interesting feedback for the specimen object of investigation can be derived from its absolute/total gradient evolution. In Figure 10b, such an absolute temperature variation is shown, as a function of time, as obtained by comparing the Exp and UnExp nodal temperatures (i.e., assuming an equivalent, fully monolithic performance for the LG cross-section). Compared to the reference limit value of 45 °C (see Table 2), the experimental cracks were typically observed to propagate for absolute thermal gradients in the order of 60–70 °C. At this stage, and up to ≈350 s of exposure, the corresponding FE model overestimates the test data in the order of 15–20%. The actual role and effect of the intermediate PVB foils for the thermal performance of the T5 nominal layered section, however, still requires further extended investigations. Figure 11, finally, presents a comparison of 1D numerical and experimental results for the 10 mm thick, MG specimen T2. For the FE assembly calibration, the same input features of the reference 1D model in Table 2 were taken into account. In the past experiment, compared to the T4 and T5 samples, the T2 specimen was exposed to a rather constant heat flux, slightly decreasing in time (see Figure 4). Such a constant loading configuration resulted in a more stable, progressive increase of temperatures in time, see Figure 11a. However, the FE results were found to overestimate the experimental predictions, for the full loading stage, on both the Exp and UnExp sides. A better numerical-to-experimental correlation was observed especially at the beginning of the thermal loading phase, see Figure 11a. Later on, however, the temperature scatter was found to lie in the range of ≈12%, which may be related to the sensitivity of FE results to the input parameters assumed in the numerical study (and in particular, the thermo-physical properties of glass), as well as to the intrinsic simplified assumptions of the 1D modeling approach.

Figure 11b, in conclusion, presents the gradient calculated for the MG specimen T2. In general, as shown, the numerical results were observed to strongly overestimate the test values, by approximately 50%. Such a constant overestimation was found for most of the Δ*T*-time history, and may be partly caused by the thermal parameters assumed for glass in the FE study, but most probably by defects of the past test measurement methods, as also described in [16]. Both the experimental and numerical temperature gradients of Figure 11b, in addition, lie below the allowable gradient of 45 °C given by standards for AN glass panels with polished edges and a nominal thickness of 10 mm (see Table 1). Such a reference limit value was found to be mostly twice the thermal gradient for the experimentally observed cracks in glass, after ≈1150 s of exposure.

### 4.2. Two-Dimensional (2D) Numerical Modelling—Reference Configuration

**Figure 12:**
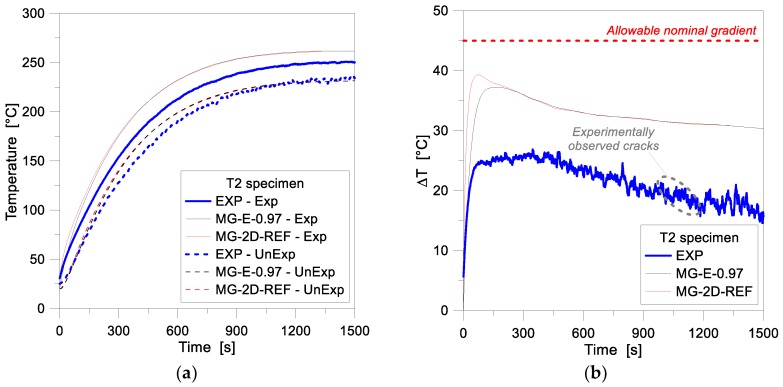
FE analyses (ABAQUS, 1D and 2D) and past experimental results for the MG specimen T2. (**a**) Temperature history and (**b**) temperature gradient comparisons.

In accordance with Section 3, selected glass samples were first analysed by accounting for 1D and 2D estimations. Figure 12, in this regard, shows a comparison of 1D-2D numerical results for the MG specimen T2, as obtained from the reference FE models of Table 2 and Table 3 at the Exp and UnExp surfaces (middle height control point, for the 2D assembly), under a uniform radiant heating exposure (see Figure 6b). In terms of temperature histories (see Figure 12a) the collected 1D and 2D results were found to be mostly identical, even if some discrepancy can be noticed at the early stage of thermal loading. There, for the first ≈200 s of exposure, the 2D model resulted in higher temperatures, with 1–2% the scatter with respect to the 1D estimations. The same phenomenon can be further noticed once the comparison of temperature gradients Δ*T* through the glass thickness is taken into account (see Figure 12b).

### 4.3. Two-Dimensional (2D) Numerical Modeling—Sensitivity Study

**Figure 13:**
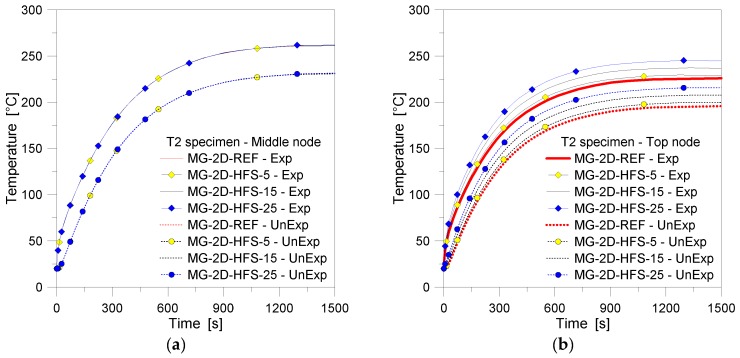
FE results (ABAQUS, 2D) for the MG specimen T2, by changing the thermal loading configuration of the sample top/bottom edges. Temperature history comparisons at the (**a**) middle and (**b**) top edge nodes.

**Figure 14:**
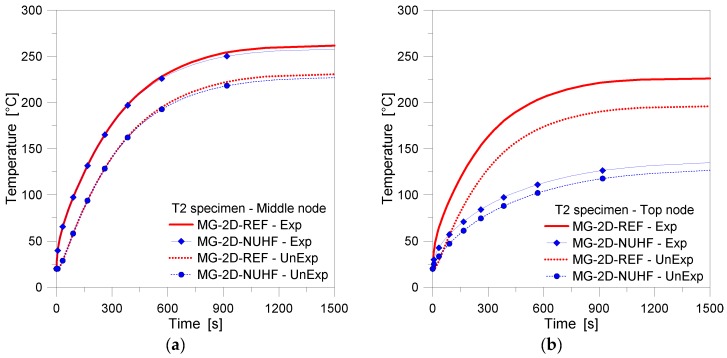
FE results (ABAQUS, 2D) for the MG specimen T2, by changing the heat flux distribution on the front surface of the glass panel. Temperature history comparisons at the (**a**) middle and (**b**) top edge nodes.

Given the FE observations briefly summarized in Figure 12, the reference 2D parametric model was hence further explored, so to assess the sensitivity of the predicted thermal responses to selected influencing parameters, according to Table 3 and Figure 6. Figure 13, for example, presents a comparison of 2D results for different loading cases, and particularly emphasizes the influence of an additional heat flux at the top/bottom edges of the FE samples (i.e., Figure 6c), with respect to the reference FE model (Figure 6b). The temperature history at the Exp and UnExp nodes in the mid-height section, in particular, shows no variations in terms of thermal response, see Figure 14a. However, marked effects due to the additional heat flux at the edges can be clearly observed in Figure 14b, as far as the mid-height control point is replaced by control points at different distances from the center of the panel. The application at the edges of a 25% the nominal heat flux, as shown, resulted in approx. a 10% increase of temperature for both the Exp and UnExp nodes. The same magnitude of temperature increase was generally calculated from the FE models under different thermal exposures on the top/bottom edges (i.e., 5% and 15% in Figure 14b), being the measured temperatures close to the top region of the FE assemblies directly proportional to the imposed heat flux amplitudes. In this regard, given the limited variations in the thermal loading scenarios of the FE models presented in Figure 14b, a high sensitivity of thermal performance estimations can be perceived, together with the intrinsic limitations of 1D models. At the same time, the 2D simulations suggest the need of a larger number of TCs for experimental performance assessments.

As far as the distribution of the imposed heat flux modifies on the overall exposed surface of glass, the FE comparisons collected in Figure 14 are achieved. There, the 2D numerical estimations derived from the FE models according to the schematic drawings of Figure 6c,d are shown. In both the FE models, an edge heat flux equal to 25% the nominal history was taken into account. As shown, the nonuniform heat flux distribution proved to have minor effects on the collected temperature histories for the FE nodes located at the middle height of the sample, see Figure 14a. Until approx. 500 s of the FE analyses, no scatter can in fact be observed between the collected curves, at the Exp and UnExp surfaces.

However, some sensitivity of FE estimations (up to ≈4% of temperature variation) can be noticed by comparing the selected numerical curves, after 600 s of thermal loading and up to the end of the analyses. Such a numerical outcome is strictly related to the fact that—at the later stages of the analyses—the heat flux that grows up at the mid-section of glass progressively moves towards less heated regions of the panel (i.e., the top and bottom edges subjected to limited thermal exposure only).

In support of this statement, Figure 14b collects in fact the temperature history at the top edge nodes, for the same FE models. As shown, a marked reduction of temperature (up to ≈43% the reference model) can be observed for the Exp node estimations, whereas a scatter in the order of ≈37% was numerically predicted from comparisons at the UnExp edge nodes.

**Figure 15:**
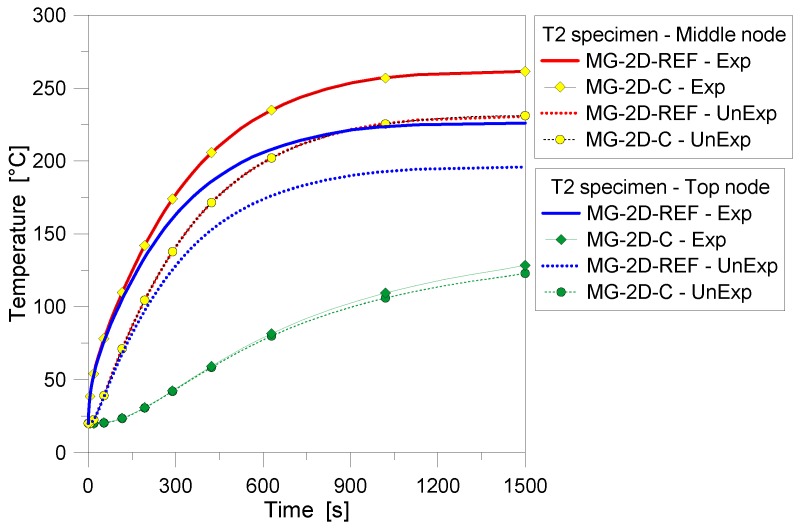
FE results (ABAQUS, 2D) for the MG specimen T2, including mechanical restraints. Temperature history comparisons at the middle and top edge nodes.

**Figure 16:**
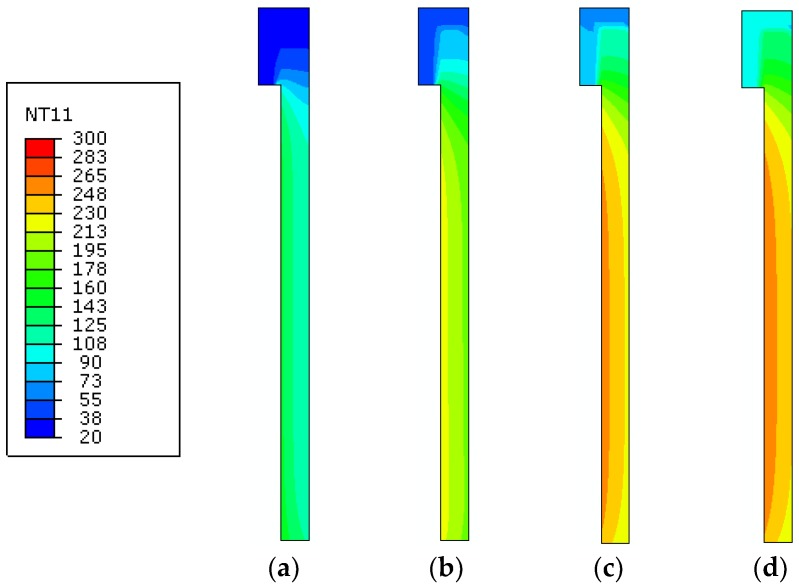
Temperature contour plots at different stages of the thermal simulation (ABAQUS, 2D), as obtained for the T2 sample with clamped edge. Temperature values are given in °C, after (**a**) 250 s; (**b**) 500 s; (**c**) 1000 s and (**d**) 1500 s of loading.

Figure 15 and Figure 16, finally, collect some further 2D results for the T2 specimen with the clamped upper edge, in accordance with Figure 6e. As shown, the presence of a partially shaded upper edge for the glass sample proved to have (apparently) minor influence on the thermal performance of the specimen, and in particular for the temperature history recorded at the middle height control point (and bottom region, in general) of the T2 panel, see Figure 15.

A more detailed analysis of the same FE results, however, gave evidence of a marked sensitivity of thermal predictions for the glass sample, especially when moving towards its top edge. In this latter case, variations up to 40% were numerically predicted, by comparing the selected FE models.

This finding is further confirmed by Figure 16, where FE results are presented for the clamped glass panel, in the form of temperature contour plots at selected time instants of thermal exposure. There, it is possible to notice how the presence of the upper clamp—even limited in dimensions—can progressively affect the temperature evolution in the glass sample, as a function of time, hence requiring careful consideration for FE modeling purposes. Special care is then expected for the overall thermomechanical analysis of the same FE assembly, due to a combination of thermal and mechanical effects, especially in the vicinity of the mechanical restraint.

At a final stage of the FE study, numerical efforts were then spent for the T5 laminated sample discussed in Figure 9 and Figure 10. Given the limited basic assumptions of the corresponding 1D model, a 2D assembly was described in accordance with Section 3, so as to assess the possible sensitivity of 1D estimations to the model accuracy. The LG shell model for the T5 specimen was implemented by accounting for the reference input features of Table 3. For the PVB layers, the thermophysical properties depicted in Figure 2a,b were considered, in accordance with [14,15]. In doing so, the mesh size of DC2D8 shell elements was still kept equal to 2 mm, with the exception of the PVB layers (0.76 mm in thickness), where two shell elements in the thickness were adopted, to ensure the consistency of thermal estimations. The final FE assembly hence resulted in ≈4400 elements and ≈13,800 DOFs.

**Figure 17:**
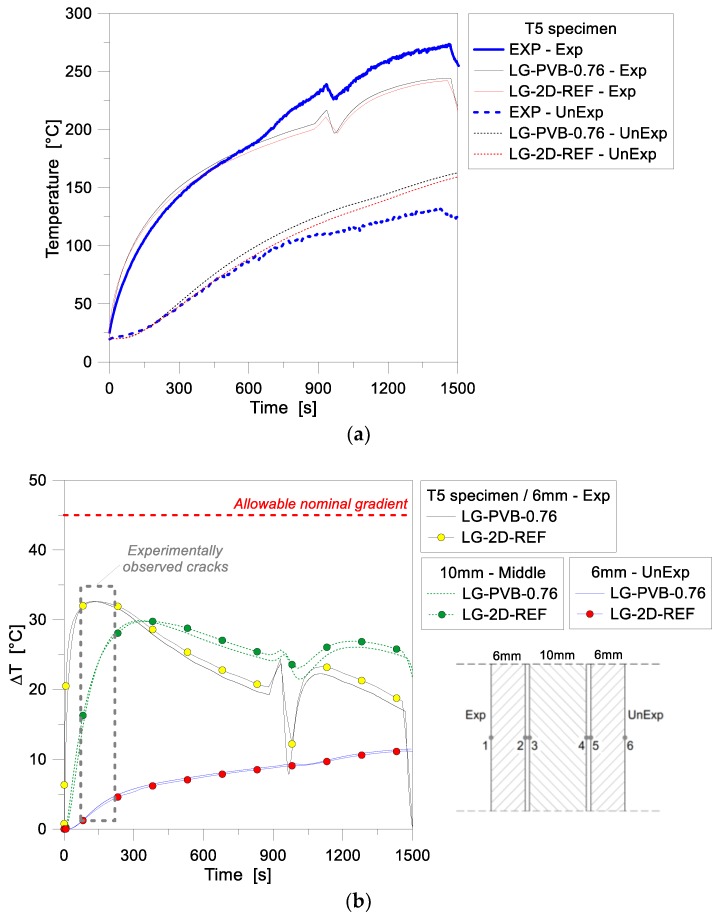
Comparative results for the LG sample T5, as obtained from the FE analyses (ABAQUS, 1D and 2D) and the past experimental test. (**a**) Temperature histories and (**b**) gradients.

**Figure 18:**
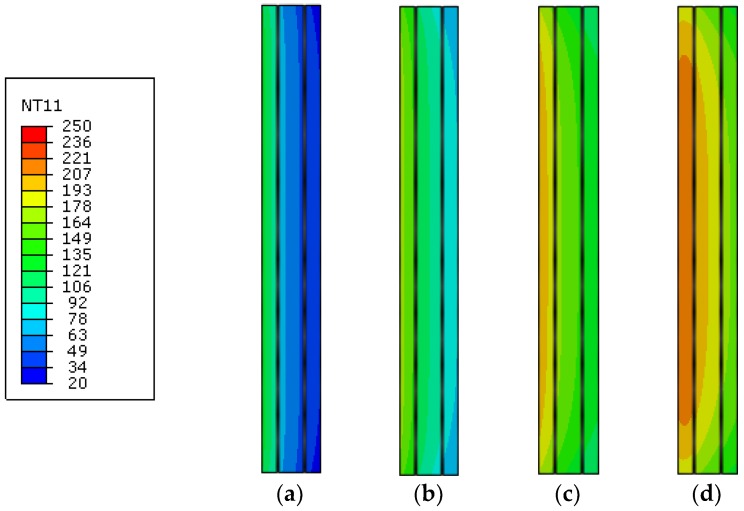
Temperature contour plots at different stages of the thermal simulation (ABAQUS, 2D), as obtained for the laminated T5 sample (PVB foils hidden from view). Temperature values are given in °C, after (**a**) 250 s; (**b**) 500 s; (**c**) 1000 s and (**d**) 1500 s of loading.

Compared to the 1D predictions, see Figure 17, the 2D model generally resulted in minor variations for the estimated temperatures, with less than 3% the average scatter. The added value of the 2D assembly, see Figure 18, is indeed represented by the temperature estimation in the full cross-section of the T5 sample, hence including possible sensitivity to cross-sectional size effects and/or edge effects, as well as (if present) mechanical restraints. From the selected contour plots of Figure 18, in particular, the progressive thermal protective contribution of the PVB foils can be perceived, as far as the temperature increases thorough the thickness of the glass panels. On the other hand—given the typical features of PVB foils—mostly negligible bonding contributions are expected from the same PVB layers, when assessing the stiffness and strength capacity of the same T5 panel under thermomechanical loads. In this sense, further extended investigations are required to explore the phenomena herein discussed.

## 5. Summary and Conclusions

In this paper, the first steps and outcomes for the development of a reliable thermomechanical FE model for structural glass systems at elevated temperatures was presented. In doing so, both 1D and 2D FE assemblies were reported and discussed, for MG and LG samples under radiant heating. Taking advantage of selected past experimental data available in the literature, in particular, comparative results and sensitivity studies were summarized in the paper, giving evidence of major issues, possible limits and influencing parameters, as well as feasibility/potential of such a modeling approach for the structural assessment of glazing systems under thermal exposure. Although the presented FE models include a number of simplifications and focus on the heat transfer phenomena only, being representative of the first key step for conventional uncoupled, thermomechanical analyses for a given structural system, several interesting conclusions were drawn from the FE numerical results and comparisons herein discussed.

One major difficulty for the FE analysis of glazing assemblies under thermal exposure, for example, is that limited information exists on the temperature dependence of various material properties, and when available, such information is typically presented for a limited range of temperatures. The use of experimental values directly derived from literature references, for example, is also difficult. In most of the cases, the specific limitations of different parameter values and/or the related empirical formulas are not clearly stated. Therefore, performing experimental tests in parallel with robust and efficient FE numerical models can be particularly beneficial.

Performing experimental tests at elevated temperature, however, includes several uncertainties and is typically time/cost-consuming. For example, even minor changes in the environmental conditions (i.e., in the testing facilities) and/or loading/boundary conditions can have noticeable effects on the collected results. In the specific case of thermal testing of glass panels and glazing systems in general, even the continuous measurement of temperatures can involve a list of challenges and uncertainties. The TCs (i.e., number and positioning within a given glass sample), their shielding from direct heat radiation and the number of wires, for example, can markedly affect and obstruct the transparency of glass specimens and represent, at the same time, local disturbances for the temperature distribution during the experiments. Therefore, in most of the cases, the relevance and accuracy of test measurements for numerical comparative purposes is not so obvious. The same applies for the heat flux measurements, since the net heat entering a specimen cannot be directly measured during radiant heating experiments.

Due to the difficulties and uncertainties herein summarized, a FE numerical approach was presented in the paper, in which—from geometrically simplified 1D heat transfer models—more detailed 2D numerical models inclusive of size and boundary effects were gradually extended. The accuracy and sensitivity of both 1D and 2D models for MG and LG glass samples was investigated, based on FE parametric studies partly summarized in the paper.

Given a generally rather good agreement between selected experimental and numerical results, the FE study also emphasized some critical aspects that should be further explored for reliable predictions on the thermal performance of glass systems. An additional step and goal of the ongoing research study, in this regard, is to further expand the refined 2D FE modeling approach, so to account for the thermomechanical behavior of glass systems under combined thermal exposure and mechanical design actions of technical interest.

## Figures and Tables

**Table 1:** Allowable temperature gradients Δ*T* for glass, according to prEN thstr:2004 recommendations [21].

	Limit Values (°C)		
Glass Type	As-Cut or Arrissed	Smooth Ground	Polished
float, or sheets ≤ 12-mm thick	35	40	45
float, 15 mm or 19-mm thick	30	35	40
float, 25-mm thick	26	30	35
patterned	26		
wired patterned or polished wired glass	22		
heat strengthened	100		
tempered	200		
laminated	smallest value of the component panes		

**Table 2:** Input properties for the 1D parametric models (ABAQUS).

Influencing Parameter	FE Model	Glass Thickness /Build-Up [mm]	Emissivity	Film Coefficient	Interlayer Thickness/Type
Emissivity & Film coefficient	MG-E-0.97 *	15	0.97	US	-
	MG-E-0.84	15	0.84	US	-
	MG-FC-8.02	15	0.84	8.02	-
Glass thickness	MG-TH-14.5	14.5	0.97	US	-
	MG-TH-15.5	15.5	0.97	US	-
Interlayer thickness	LG-PVB-0.76	6 + 10 + 6	0.97	US	0.76 mm/PVB
	LG-PVB-1.52	6 + 10 + 6	0.97	US	1.52 mm/PVB

US = user subroutine; * = reference 1D model for MG samples.

**Table 3:** Input properties for the 2D parametric models (ABAQUS).

FE Model	Heat Flux **			
	Exposed (Front) Surface	Top/Bottom Surface	Mechanical Restrains	Figure
MG-2D-REF *	100%	-	-	6b
MG-2D-HFS-5	100%	5%	-	6c
MG-2D-HFS-15	100%	15%	-	6c
MG-2D-HFS-25	100%	25%	-	6c
MG-2D-NUHF	100%/25%	25%	-	6d
MG-2D-C	100%	25%	Yes (steel clamp)	6e

* = reference 2D model; ** = imposed heat flux amplitude, compared to the nominal curves of Figure 4.

## List of Abbreviations

The following abbreviations are used in this manuscript (list in alphabetical order):

AN	Annealed (glass)
CFD	Computational Fluid Dynamics
DOF	Degree Of Freedom
Exp	Exposed (node)
EXP	Experimental
EVA	Ethylene Vinyl Acetate
FE	Finite Element
FT	Fully Tempered (glass)
GFRP	Glass-Fiber-Reinforced Polymer
LG	Laminated glass
MG	Monolithic glass
MOE	Modulus of Elasticity
HS	Heat strengthened (glass)
PVB	PolyVinyl Butyral
SG	SentryGlas^®^
SLS	Soda-Lime-Silica (glass)
TC	Thermocouple
TPS	Transient Plane Source
UnExp	Unexposed (node)
1D	one-dimensional
2D	two-dimensional
